# Sustainability of the Linkages Between Water–Energy–Food Resources Based on Structural Equation Modeling Under Changing Climate: A Case Study of Narok County (Kenya) and Vhembe District Municipality (South Africa)

**DOI:** 10.3390/su16229689

**Published:** 2024-11-07

**Authors:** Nosipho Zwane, Joel O. Botai, Christina M. Botai, Tafadzwanashe Mabhaudhi

**Affiliations:** 1 South African Weather Service, Private Bag X097, Pretoria 0001, South Africa; 2 Department of Geography, Geoinformatics and Meteorology, University of Pretoria, Private Bag X020, Hatfield, Pretoria 0028, South Africa; 3 Centre on Climate Change and Planetary Health, London School of Hygiene and Tropical Medicine, London WC1E 7HT, UK; 4 Centre for Transformative Agricultural and Food Systems, School of Agriculture, Earth and Environmental Sciences, University of KwaZulu Natal, Pietermaritzburg 3209, South Africa; 5 United Nations University Institute for Water, Environment and Health (UNU-INWEH), Richmond Hill, ON L4B 3P4, Canada

**Keywords:** WEF resources, climate change, structural equation modeling, sustainability

## Abstract

Due to the current and predicted increase in the global demand for water–energy–food (WEF) resources, as well as the inevitable linkages between the WEF sectors and sustainable development, the WEF nexus is rapidly being recognized as a method to effectively manage sustainable development. Many African countries still face challenges in terms of the demand for and accessibility of WEF resources. For this reason, a comparative study of two sites (Narok County and Vhembe District Municipality), which exhibit similar socio-economic, environmental, and technological circumstances, was undertaken. In the present study, we considered 218 questionnaire responses, which we analyzed using partial least squares structural equation modeling (SEM) based on the WEF nexus constructs. This study is anchored on the null hypothesis (H0), whereby no interdependencies exist between the state of the climate and WEF resources, as constrained by sustainable development options. The results show that the proposed hypothesis does not hold, but rather, an alternative hypothesis (Ha)—there exist linkages between climate change and WEF resources—holds. This is demonstrated by the descriptive statistics indicating *p* values < 0.05 for both the *t*-test and the Bartlett test. Furthermore, analysis from the multi-regression, particularly for the model where we combined the sites, showed *p* values < 0.05 and higher adjusted r-squared values, which denoted a better fit. The communities in both study sites agree that the regions have experienced a scarcity of WEF resources due to climate change. The results show that climate change is an intrinsic part of the developmental options for the sustainable livelihood of both study sites, which aligns with the 2030 UN agenda on sustainable development goals targets. Moreover, the sustainable management of natural resources that are people- and planet-centric is crucial to climate change adaptation and mitigation, social justice, equity, and inclusion. The SEM results showed with significant confidence that the water, energy, and food sectors are closely interconnected; however, their impact on climate and sustainability is significantly different. Food has a direct positive impact on climate and sustainability, while both water and energy have an indirect negative impact. Moreover, the climate construct indicated a significant direct link to sustainability for all the relationships explored. This is particularly true because, in most underdeveloped countries, sustainable development and societal wellbeing heavily rely on goods and services derived from natural resources and the environment. This study contributes to the nexus modeling research field by introducing SEM as an innovative methodology over a single equation modeling framework in analyzing variables that have complex interrelationships, facilitating advanced WEF nexus resource governance.

## 1 Introduction

Major global concerns, including climate change, environmental degradation, migration, population increase, and fast urbanization, require integrated approaches to effectively manage resources and guarantee sustainable accessibility and availability [1,2]. According to studies by Adom et al. [3] and Liu et al. [4], integrated solutions call for stakeholder buy-in and public awareness from the onset as they require a paradigm change from the typical “silo” approach to a cross-cutting one that recognizes and encourages cross-sectoral convergence and coherence in resource management. The nexus of water, energy, and food (referred to as the WEF nexus) is increasingly being recognized as a strategy to efficiently manage sustainable development due to the current and anticipated growth in global demand and pressure on WEF resources, as well as the strong links between the WEF sectors and sustainable development [3,5,6].

The WEF nexus is intricate yet critical to resolve many of humanity’s problems [3,4,7]. The WEF nexus is three-dimensional—(1) it is utilized as a conceptual framework, (2) as an analytical tool, and (3) as a discourse. A study by Nhamo et al. [8] suggested that the conceptual framework makes it easier to understand how WEF connections support coherence in policymaking and advance sustainable development. Meanwhile, as an analytical tool, it systematically applies quantitative and qualitative methods to understand the interactions between WEF resources. Lastly, as a discourse, it is a tool for problem-framing and fostering cross-sectoral collaboration.

Many African countries still have significant disparities in demand and accessibility, implying that millions of people lack water, energy, and food resources [9]. The three resources are strongly linked, and any impact on one affects the others. A WEF nexus approach could unlock the synergies required to prompt regional socio-economic development. The necessity for a coordinated and integrated approach to sustainable development, like the WEF nexus model, is further justified by climate change estimates showing greater demand for water, energy, and food resources [5,10]. An improved integrated management of the WEF sectors has the potential to substantially improve climate resilience at local, national, and regional levels [11].

According to Thuo et al. [11], Kenya foresees becoming a “middle-income country providing a high quality of life to its citizens by 2030”. Kenya Vision 2030 also includes the WEF sectors. Regarding energy, Kenya wants to produce more energy at a cheaper cost and consume it more efficiently. The government is committed to institutional reforms, including increasing the number of independent power producers and exploiting new sources, including geothermal, coal, and renewable energy sources. In terms of agriculture, Kenya aims to increase revenues in agriculture, livestock, and fishing by adding value to the products before they are sold. This involves using 1.2 million hectares of newly opened land and 1 million hectares of existing uncultivated land. Lastly, Vision 2030 recognizes that Kenya is a water-scarce country and suggests building two large and 22 medium-sized dams and renovating a few irrigation projects. In Narok County, both livelihoods and WEF resources are threatened by climate change. Rural households in Narok depend on various activities and income bases, and crop and livestock production are prominent [11–14].

Similarly, in South Africa, WEF resources are constrained. The literature shows that access to water, energy, and food is crucial for securing fundamental human rights and dignity. It is central to global sustainability challenges, making it necessary to manage and allocate the available resources sustainably and in an integrated manner [3,15]. In Vhembe District Municipality (VDM), the community relies on dirty open-water reservoirs like rivers and dams due to a lack of reliable water resources. The district’s water security is also in jeopardy due to the rise in mining operations, which consume significant amounts of water. About 64% of homes rely on wood and other cheap energy sources, while only 36% have access to electricity. Rain-fed agriculture is the primary source of income in households that are engaged in agriculture [16–18].

The present study seeks to apply structural equation modeling (SEM) methodology to explain the explicit or implicit impacts of climate change on WEF resources in Narok County (Kenya) and Vhembe District Municipality (South Africa), with the main aim being to explore the synergies between WEF resources and driving factors. Moreover, this study intends to distinguish the direct and indirect effects of WEF security. Additionally, the specific objectives of this study include (a) the assessment of the WEF resources across the study sites, (b) conceptualizing a structural equation model to mimic the interlinkages between climate change drivers and WEF resources, and (c) the proposal of a scientific and practical contribution of the WEF nexus research paradigm from the perspective of SEM. The study sites are defined as rural with low economic activity, and the population depends on natural resources—mainly rainfed agriculture. The contemporary megatrends in society, in conjunction with shifts in the environment, technology, economy, politics, and demographics, continue to strain the limited and diminishing natural resources, endangering their sustainability and, in turn, weakening the resilience of communities.

## 2 Materials and Methods

The analysis is based on qualitative mapping of the interlinkages between the water, food, and energy systems using data collected from questionnaires, focused group discussions, and observations from the field when we visited the study areas. The relevant literature downloaded from Web of Science and Scopus was also considered. Using R studio (version 4.3.3), the survey responses were analyzed for distribution and descriptive statistics, where the Student’s *t*-test was used to compare the mean between responses of the two study sites. The Bartlett test also tested equal variances in responses from Kenya and South Africa. In this regard, if *p* ≤ 0.05, the chi-squared test is considered significant; then, the null hypothesis is rejected, and consequently, the variances are assumed to be unequal. Furthermore, we considered the multi-regression analysis, in which we investigated the data from the two sites against the independent variables (i.e., exposure and nutrition).

A WEF matrix was formulated from the literature and survey responses, which informed sustainable WEF resource interdependencies in the study sites. Moreover, we defined various factors that act as drivers and affect the intricate WEF nexus system using the SEM. For the SEM, a partial least squares path model was employed. The SEM focuses on the indirect (mediated) and direct effects of variables on other variables; the reliability analysis was conducted to validate the interrelationships between the constructs. The current study was framed on the null hypothesis (H0): No interdependencies exist between the state of the climate and the WEF resources as constrained by the sustainable development options.

**Figure 1:**
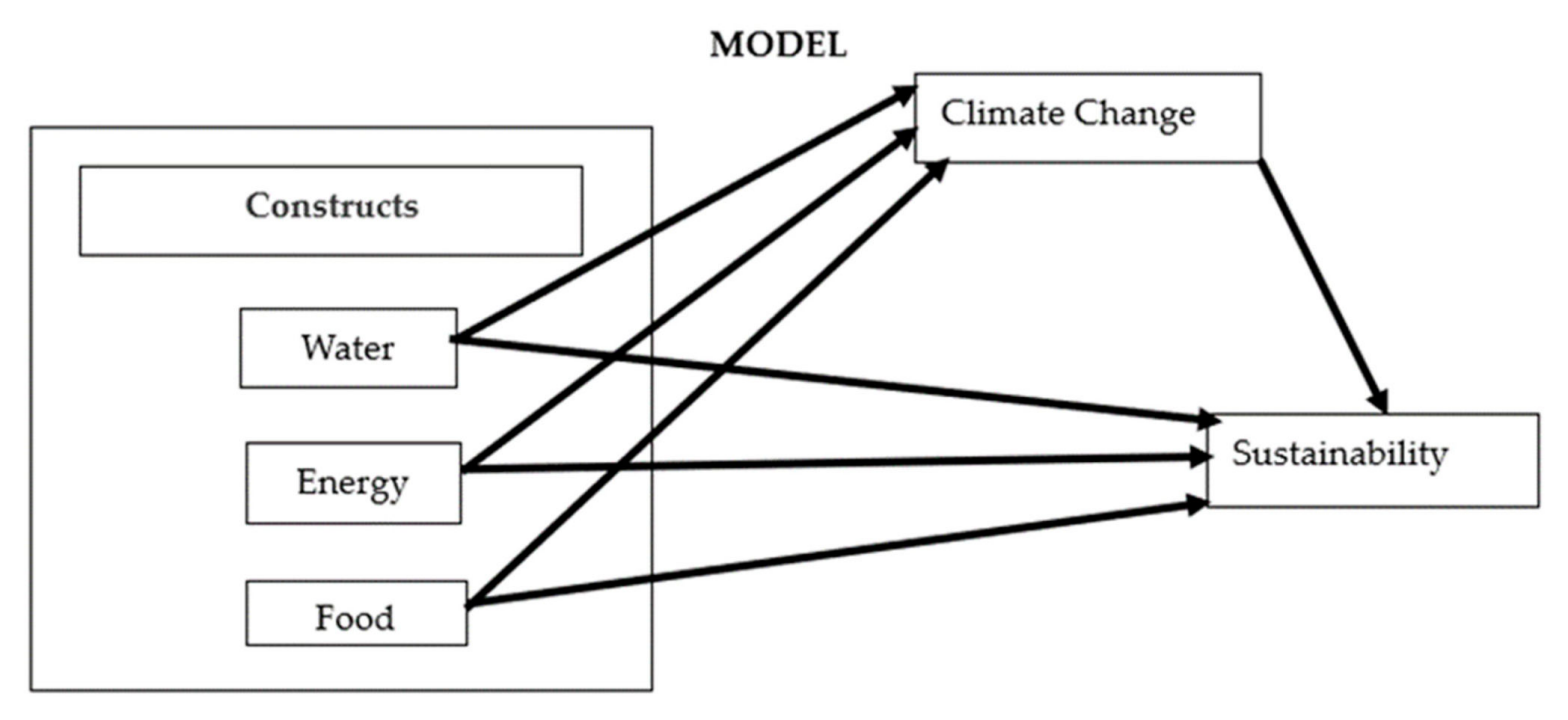
Hypothesized structural equation model.

Appendix A defines the variables considered for analysis from the questionnaire. Figure 1 shows the hypothesized model, where water, energy, and food are the constructs, the independent variable is climate change, and the dependent variable is sustainability.

## 3 Results and Discussion

### 3.1 Questionnaire Descriptive Statistics

Table 1 shows the descriptive statistics for the *t*-test and the Bartlett test in Narok County and Vhembe District Municipality and the results for the combined data for the two study sites. The Student’s *t*-test and the Bartlett test showed that the *p*-values were less than 0.05; therefore, they were significant with a 95% confidence interval that the alternative hypothesis is true: there exist interdependencies between the state of the climate and WEF resources.

Table 2 illustrates the multi-regression analysis where three models involving contrast variables defined as climate change, sustainable development, and community recovery were tested against the independent variables with significance exposure and nutrition. The model shows significant variations within the data for Narok County, with *p*-values less than 0.05. The opposite is observed for the Vhembe District Municipality, where the model shows variation but is not statistically significant. The combined data for both the study sites give a clearer picture, showing that the contrast and independent variables are statistically significant for all model variations with *p*-values < 0.05 and higher adjusted r-squared values. Therefore, we can reject the null hypothesis. This means that the alternative hypothesis is true: interdependencies exist between the state of the climate and WEF resources.

Table 3 shows the results for the multi-regression analysis for the combined constructs to explain why using linear models is not feasible for complex interrelationships. As shown in Table 3, while the constructs and independent variables show dispersed statistically significant linear correlations, the individual constructs have statistically significant linear correlations (i.e., the *p*-values were all <0.1). As a result, the present study considered the SEM framework a feasible methodology to analyze variables with complex interrelationships.

### 3.2 Questionnaire Distribution Statistics

**Figure 2:**
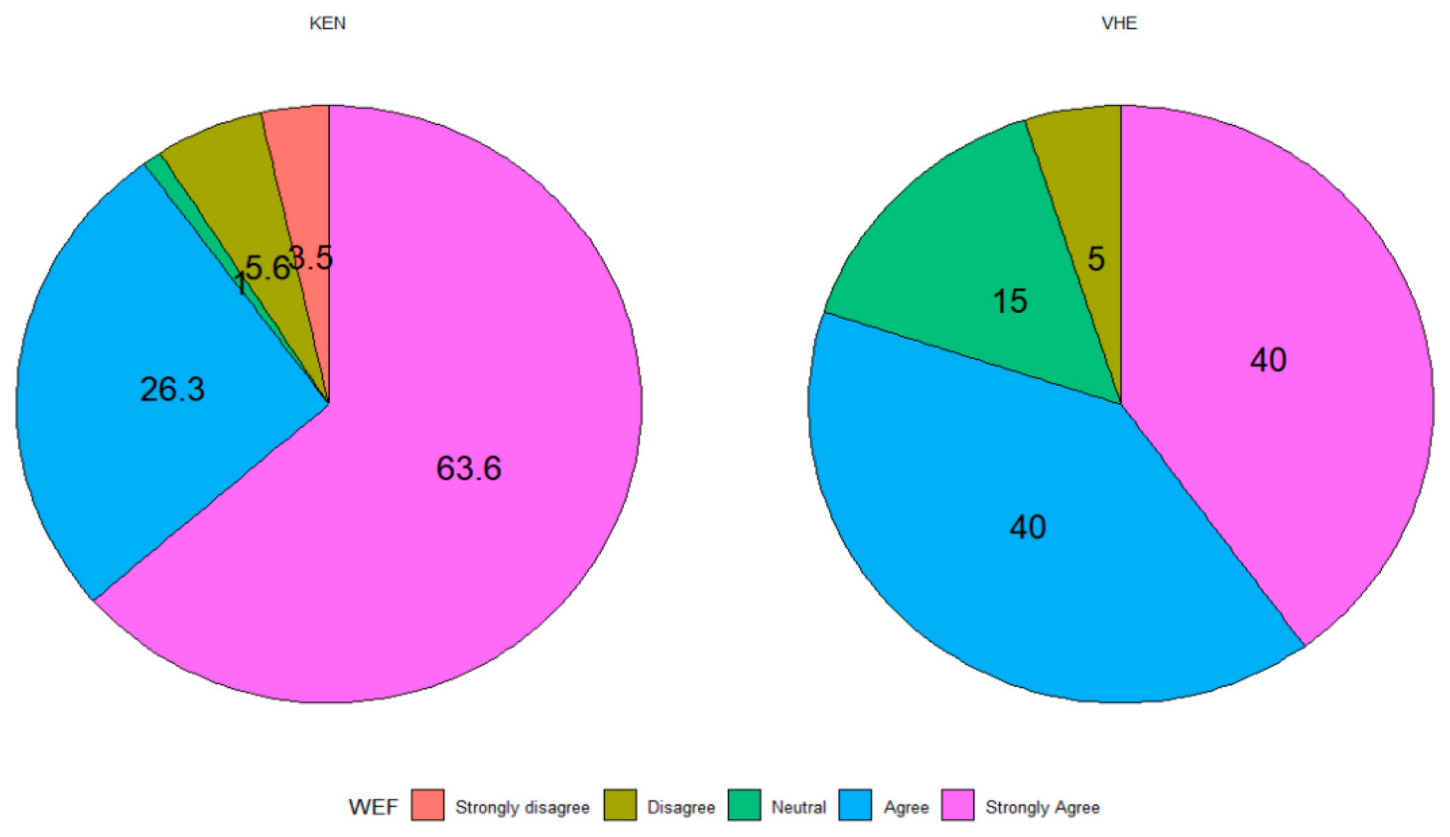
Distribution of responses in the study sites (the values are in percentages).

The distribution of responses from Narok County and VDM on the question “Does the community or region experience scarcity of water, energy, or food resources due to climate changes?” is shown in Figure 2. In Vhembe, 40% of the respondents strongly agreed, and another 40% agreed. A total of 15% were neutral, while only 5% disagreed. A slightly different response was observed for Narok County, where 63.3% strongly agreed, and 26.3% agreed. About 1.3% were neutral, and 5.6% and 3.5% disagreed or strongly disagreed. The results in Figure 2 illustrate that communities in both the study areas agree that the regions have experienced a scarcity of WEF resources due to the changing climate.

**Figure 3:**
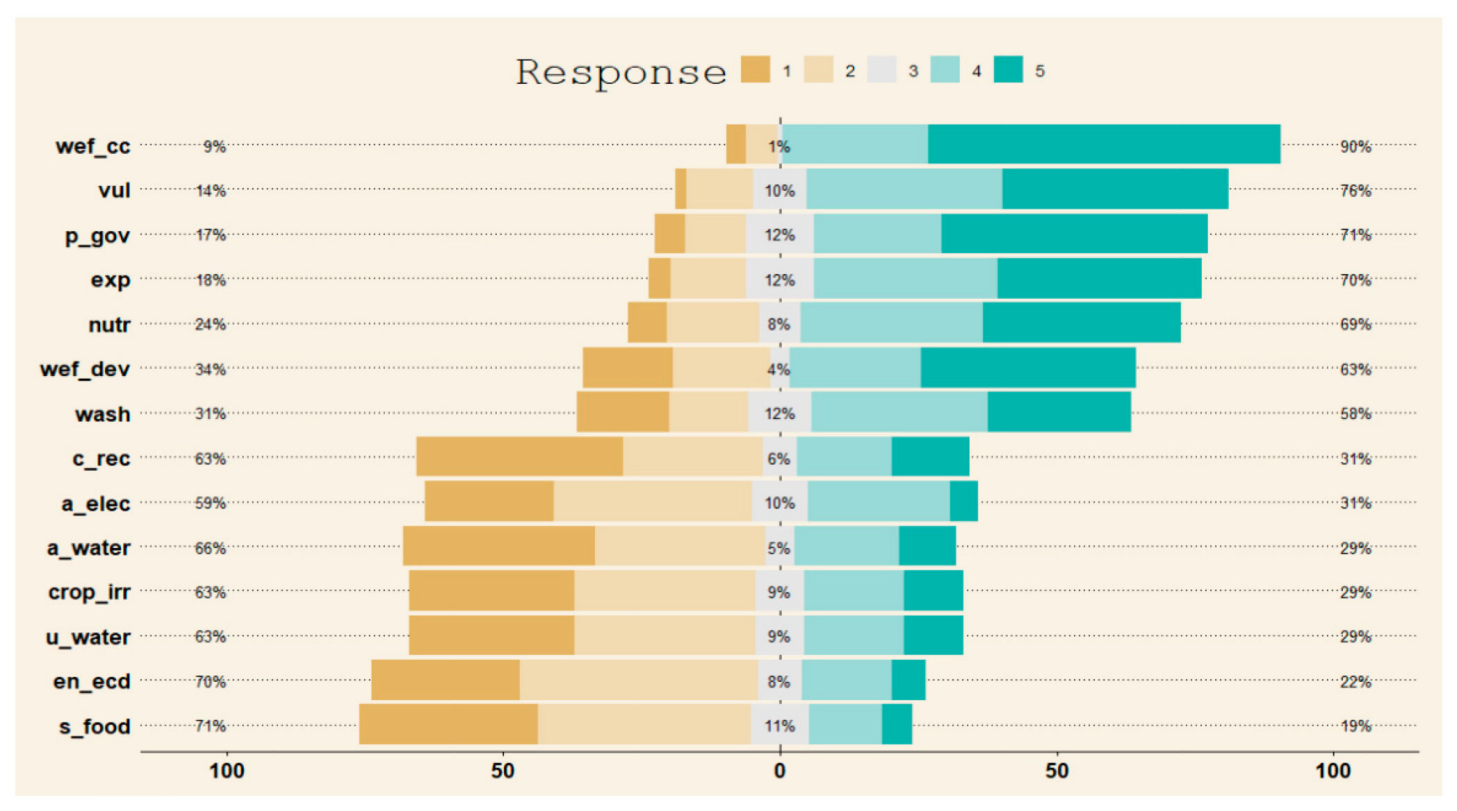
Combined distribution of quantitative survey responses from both study sites.

Figure 3 depicts the combined distribution of survey responses from Narok County and Vhembe District Municipality across all the variables listed in Appendix A—which assesses sustainable livelihood, health, and wellbeing in the communities. Demonstrated in Figure 2 is that the questionnaire was well-structured. Most respondents had an opinion about the statements, and only a small percentage of respondents were neutral (grey). On the statements that looked at WEF resources, climate change, vulnerability, governance, exposure, nutrition, development, and water—sanitation and hygiene, the respondents generally agreed/strongly agreed with a rating scale of 4/5, which is indicated by a percentage range of 58–90% (green). Meanwhile, statements that looked at WEF resources, recovery, access to electricity, access to water, crop irrigation, economic development, and food security, the respondents generally strongly disagreed/disagreed with a rating scale of 1/2 indicated by a 63–71% range (brown).

**Figure 4:**
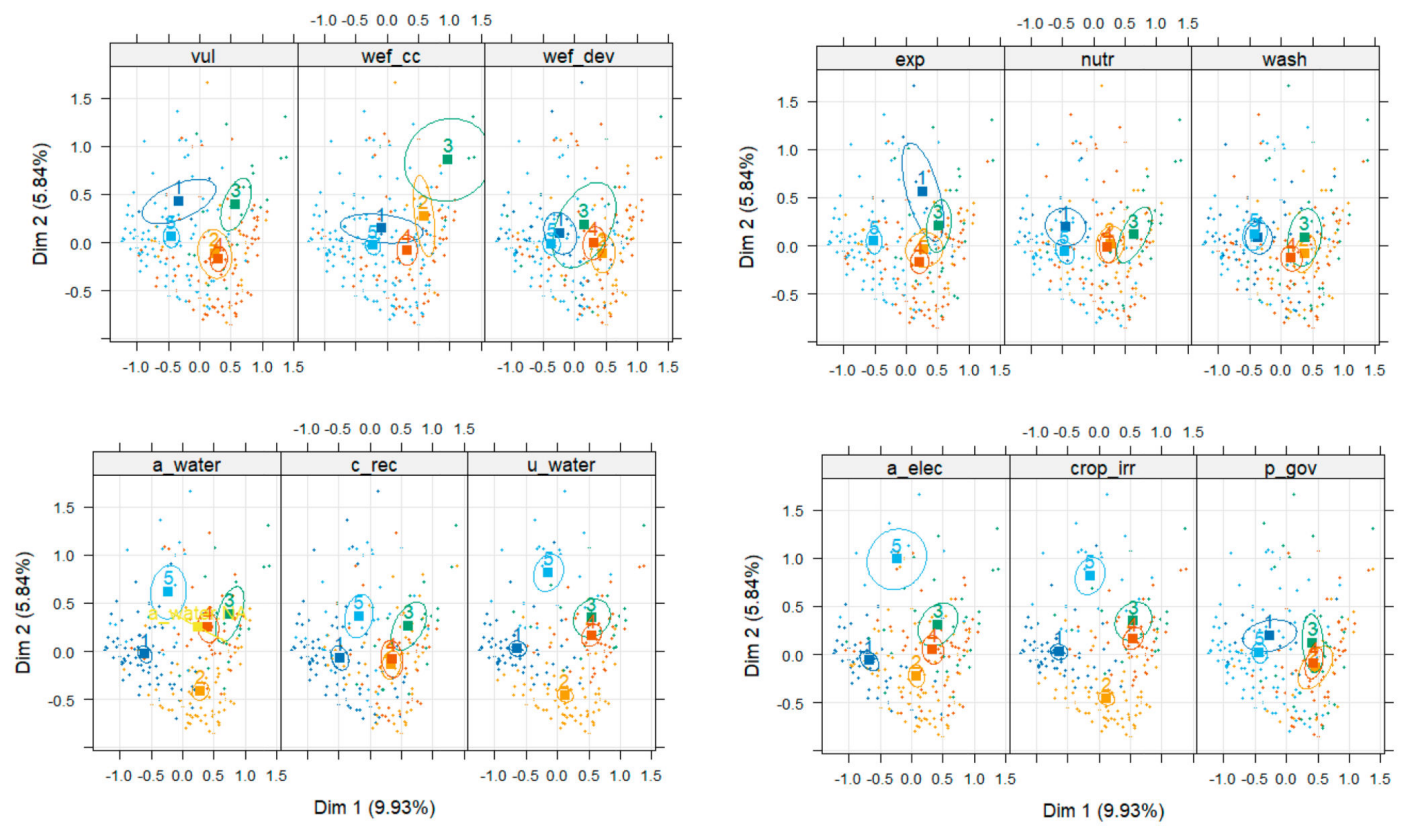
Multiple correspondence analysis draws confidence ellipses around the categories of all variables used.

Figure 4 shows the confidence ellipses around the categories of the selected variables for both study sites. Illustrated in this figure are the categories that are different from each other. Responses from both the study sites indicate that the variables that are not significantly different agree regarding how climate change impacts sustainable livelihoods, health, and wellbeing in the communities. These included vulnerability, exposure, governance, climate change, water, sanitation, and hygiene.

The WEF nexus approach investigates interconnections between various WEF sectors, often considered to have synergies and trade-offs. A cross-sectoral approach to the WEF sectors offers an opportunity to achieve positive synergies and efficiently handle trade-offs [19,20]. The WEF nexus approach could play a major role in achieving the sustainable development goals (SDGs) of the United Nations, particularly goals 2 (zero hunger), 3 (good health and wellbeing), 6 (clean water and sanitation), 7 (affordable and clean energy), and 13 (climate action) [19,20].

This study formulated two hypotheses: (1) null hypothesis (H0)—there exist no inter-dependencies between the state of the climate and the WEF resources, and (2) alternative hypothesis (Ha)—there exist interdependencies between the state of the climate and WEF resources. the results of this study show with a significant 95% confidence interval that the alternative hypothesis is true, as demonstrated by the descriptive statistics indicating *p*-values < 0.05 for both the *t*-test and the Bartlett test. Furthermore, analysis from the multi-regression, particularly for the model where we combined the sites, showed *p*-values < 0.05 and higher adjusted r-squared values, which denotes a better fit. The results demonstrate that climate change is an integral part of developmental options for the sustainable livelihood of both study sites, as opined in [19]. Furthermore, the findings corroborate Bhaduri et al. and Zaman et al. [5,10] that water, energy, and food, as well as the environment and health, are central to sustainable development, because these factors are essential for the socio-ecological and socio-economic sustainability and the wellbeing of people and the environment.

### 3.3 Structural Equation Model

**Figure 5:**
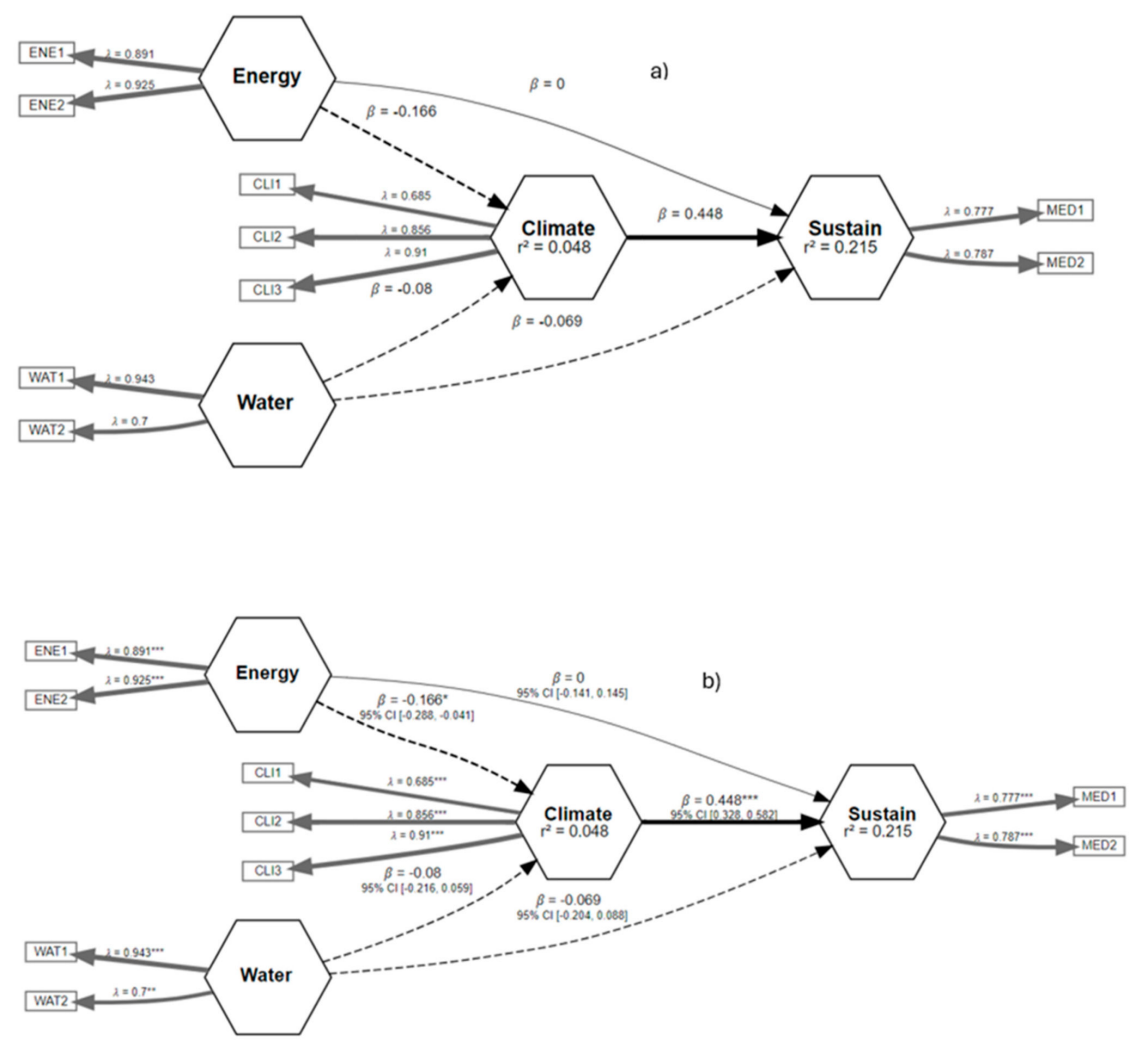
Relationship 1—Water, energy, climate, and sustainability model shown in (**a**) and the bootstrapped model in (**b**). Asterisk is the significance level. * *p* < 0.05 ** *p* < 0.01 *** *p* < 0.001.

**Figure 6:**
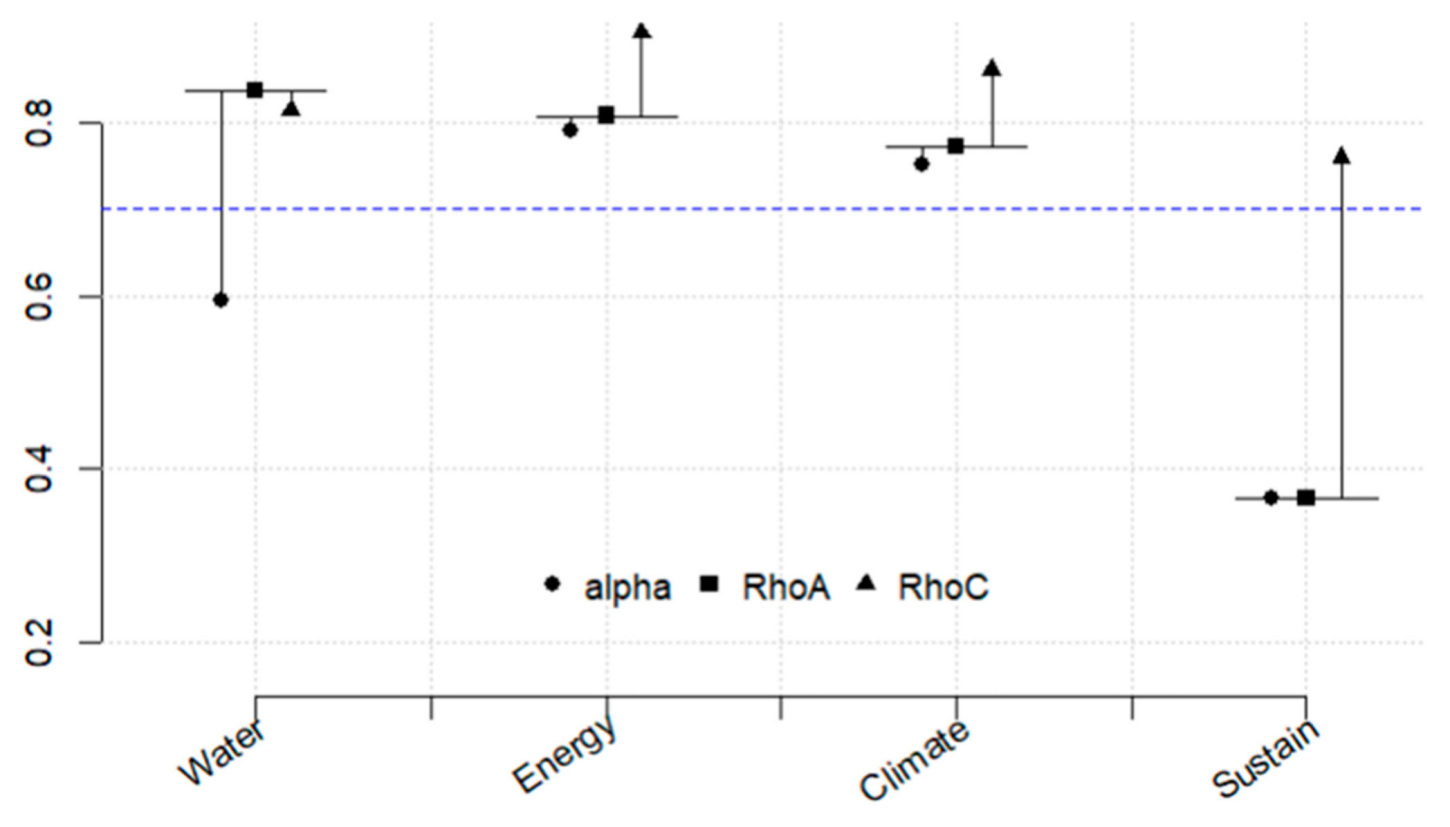
Reliability graph for relationship 1—Water, energy, climate, and sustainability. The blue dashed line is the threshold value for reliability metrics.

**Figure 7:**
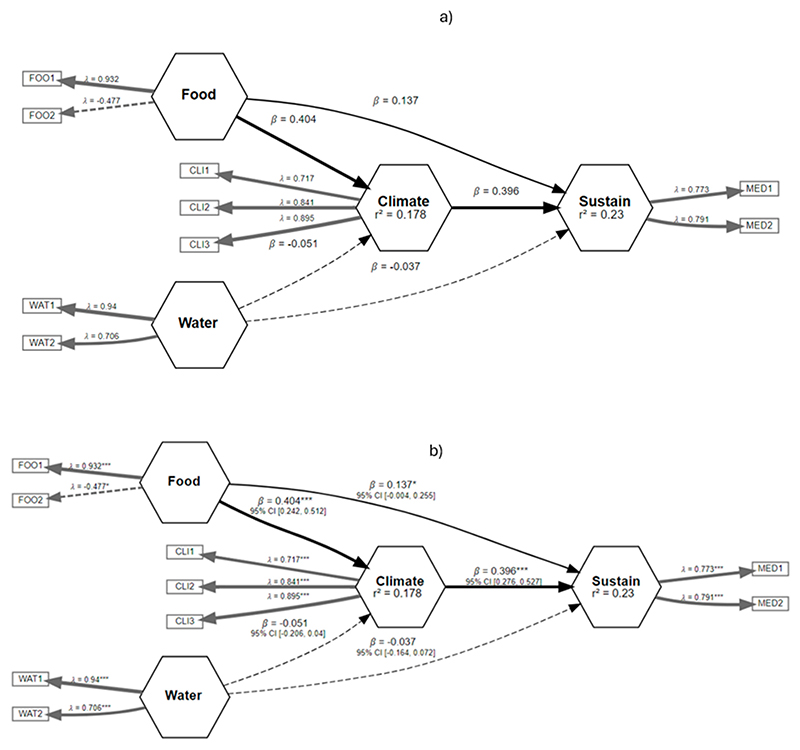
Relationship 2—Water, food, climate, and sustainability model shown in (**a**) and the bootstrapped model in (**b**). Asterisk is the significance level, see footer in Table 3.

**Figure 8:**
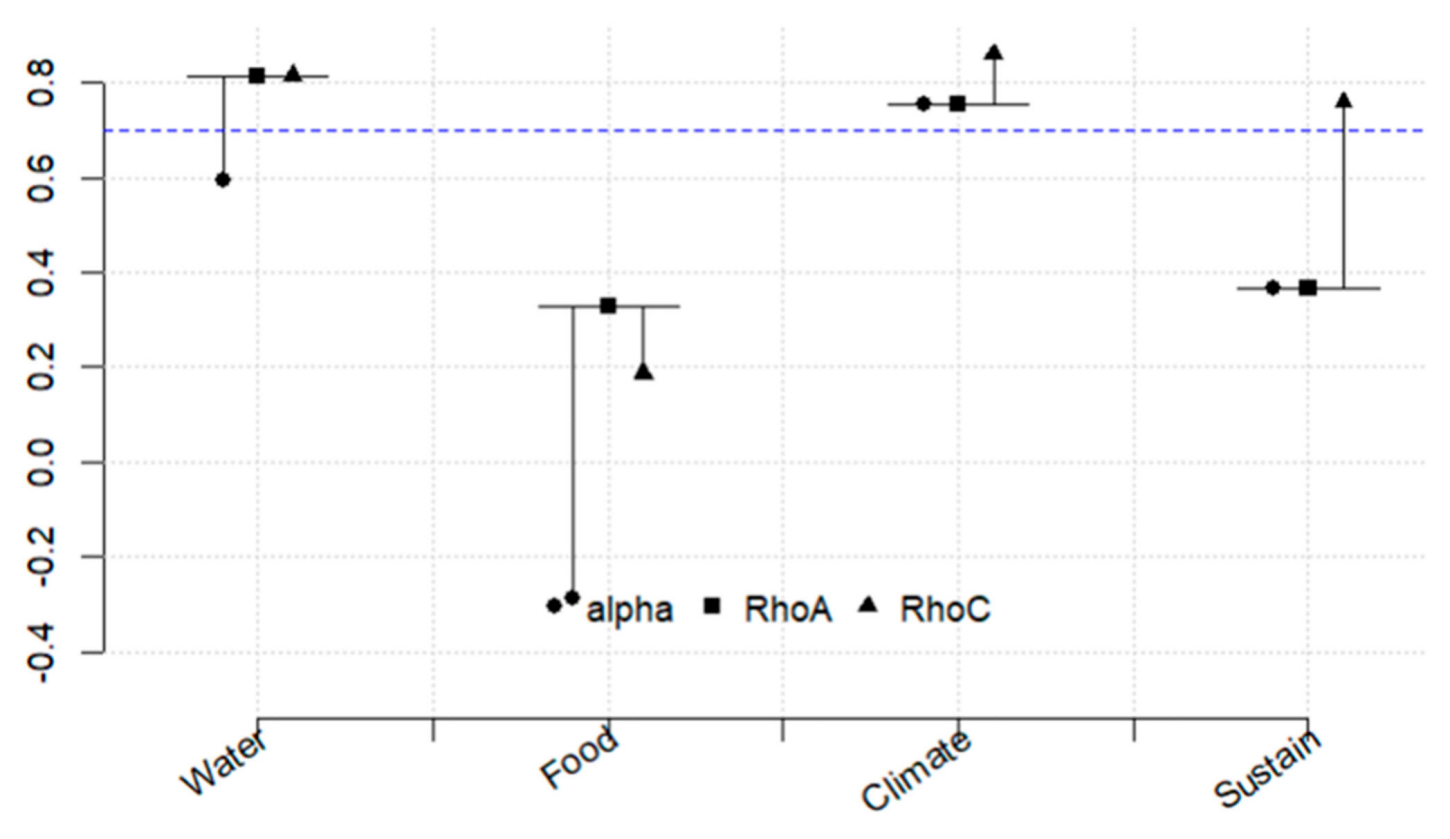
Reliability graph for relationship 2—Water, food, climate, and sustainability.

**Figure 9:**
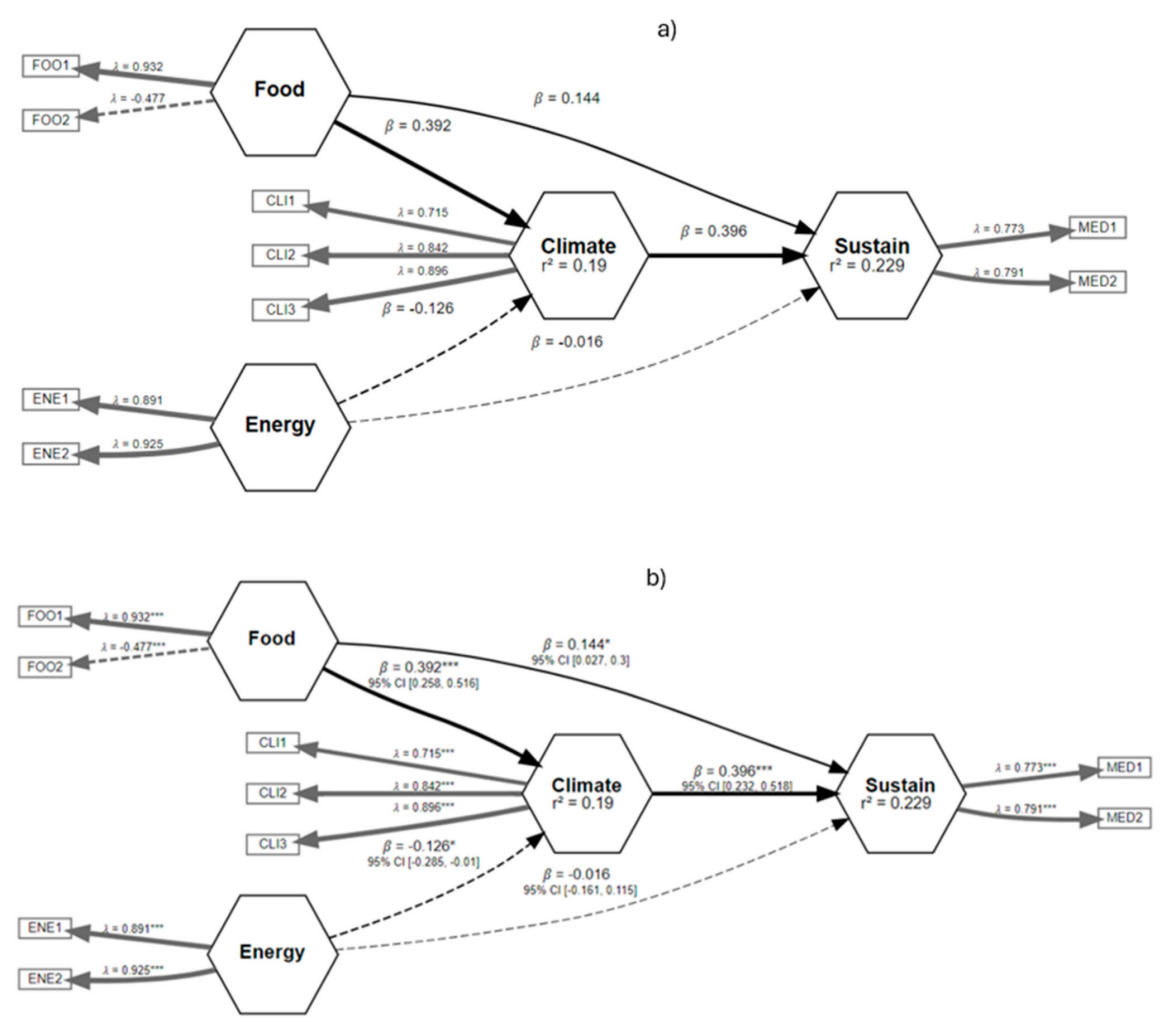
Relationship 3—Energy, food, climate, and sustainability model shown in (**a**) and the bootstrapped model in (**b**). Asterisk is the significance level, refer to the footer in Table 3.

**Figure 10:**
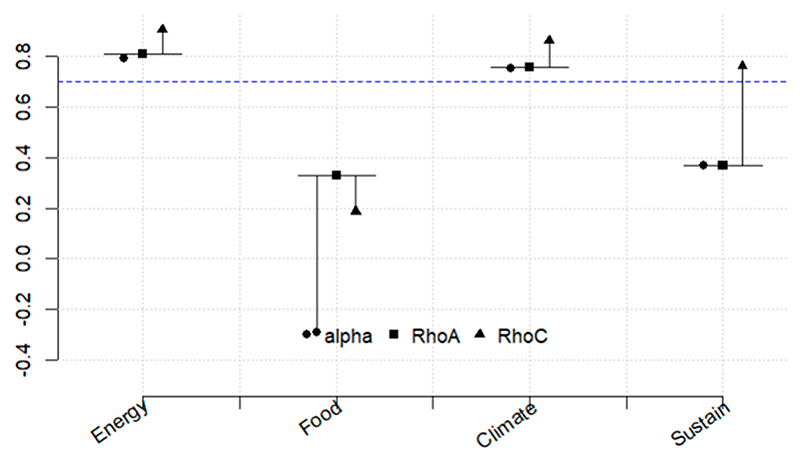
Reliability graph for relationship 3—energy, food, climate, and sustainability.

Figures 5–10 illustrate the three relationships formulated and investigated using the SEM framework. Relationship 1 comprises water, energy, climate, and sustainability. Relationships 2 and 3 correspond to water, food, climate, and sustainability and food, energy, climate, and sustainability, respectively. Figures 5–10 show the output of the original model versus the bootstrapped model and a reliability graph (e.g., a diagram that summarizes and evaluates the relationships/properties of the selected variables) for both the Narok County and Vhembe District Municipality sites. For the bootstrapped model, we calculated a 5% confidence interval for the mediated path (i.e., water–energy–food–climate–sustainability) and a 5% confidence interval for the direct path (i.e., climate–sustainability), as highlighted in Table 3. The bootstrapped model output also indicates the significant and non-significant levels within the relationships. Water, energy, food, and sustainability have two measured observed variables, while climate has three. In addition, from the reliability graph, we show the confidence and validate the model accuracy, where Alpha, rhoC, and rhoA should exceed a threshold of 0.7. Furthermore, Table 4 provides the standard deviations (SD) of the mediation paths of the constructs: water, food and energy (independent variables) and sustainability (dependent variable).

Figure 5 demonstrates relationship 1, where both energy and water have an indirect effect on climate. The negative impact for energy is significant with a coefficient of β = − 0.166 *** (asterisk is the significance level). A direct effect is observed from energy to sustainability, and an indirect effect is observed from water to sustainability. Moreover, a significant positive impact from climate to sustainability is evident with a coefficient of β = 0.448 ***. In relationship 1, water, energy, and climate show more reliability, as demonstrated in Figure 6.

Relationship 2—water, food, climate, and sustainability are demonstrated in Figure 7. Food has a significant positive direct impact on both climate and sustainability. The significance level is more from food to climate than from food to sustainability, indicated by the coefficient of β = 0.404 *** and β = 0.137 *. Similar to relationship 1, climate is also directly linked to sustainability. Unlike food, water indicates an indirect link to climate and sustainability with a coefficient of β = − 0.051 and a coefficient of β = − 0.037, respectively. Water and climate show more reliability in this model, and food is the least reliable, as illustrated in Figure 8.

Lastly, relationship 3 is explored in Figure 9, where food is directly linked to climate and sustainability. In contrast, energy depicts an indirect negative impact on both climate and sustainability. The negative impact is significant from energy to climate with the coefficients of *β* = − 0.126 *. Climate change significantly affects sustainability with a positive impact factor coefficient *β* = 0.396 ***. The reliability graph shows that both energy and climate are reliable.

The SEM framework does not determine causation between the variables [20]; however, in this study, the SEM model showed with significant confidence that the water, energy, and food sectors are closely interconnected under a changing climate and sustainability, but differently. The negative impacts influencing the three sectors mostly manifest from the increased stress and scarcities of the WEF resources, complimented by a high rate of population growth, variations in resource consumption, including land-use patterns, and climate change [21]. The current findings are supported by numerous studies reported in the literature, mostly emphasizing the urgent need to focus on the sustainability of the WEF nexus and the implementation of effective management plans that support an enhancement in WEF resource security [3–5,19]. Moreover, the results showed that food security is directly impacted by changing climate, whilst it is interlinked to sustainability, a pre-condition for long-term food security. According to the current findings, studies by Meybeck et al. [22] found a direct impact of climate change on food production and distribution, with changing precipitation patterns being the main contributing factor to reductions in crops and agricultural yields [23]. Furthermore, water and energy have indirect negative impacts on sustainable development. Such impacts are still fundamental given that water and energy contribute towards poverty alleviation and economic development, thus supporting the sustainable development goals.

Considering all the relationships explored in this study, climate change is directly linked to a community’s health, wellbeing, and sustainable livelihoods, as proffered in the well-documented SDGs. The World Meteorological Organization (WMO) reported a new record for daily temperatures [24] wherein the period from June 2023 to June 2024 indicated the warmest conditions compared to recent years. South Africa and Kenya, like many African countries, are the regions most vulnerable to the impacts of climate change. Various studies have alluded that the frequency and magnitude of extreme weather and climate events in both countries are rising faster, overtaking the population capacity to manage the supply of key resources (e.g., water demand exceeding supply) and weather-related disasters [25–28]. In addition, the global warming trend is expected to exacerbate extreme weather events’ severity, frequency, and duration [29], such as heatwaves, droughts, floods, and thunderstorms. Consequently, extreme weather and climate events will likely threaten socio-economic and environmental wellbeing and sustainable development [30].

The report from WMO paints a clear picture that climate has a direct impact on sustainability. Our analysis shows that efforts in one sector alone cannot improve WEF security and that external factors may disturb the WEF systems. Consequently, WEF security could be effectively ensured when improving indicators in every field with the highest influence. A cross-sectoral, cohesive, and integrated perspective is embraced by solutions that use a WEF nexus approach, representing a fundamental shift from conventional sectoral approaches [19]. Reaching the Sustainable Development Goal targets for Goals 2, 6, and 7 (food, energy, and water) is essential to achieving the global aspirations for a sustainable future, which include planetary health and resource security [2,3]. To accelerate progress towards achieving the SDGs, transformative, cross-sectoral, and circular approaches that improve resource use efficiency and sustainability must be adopted [3]. Thus, recognizing and appreciating the interlinkages between WEF resources is the first step towards achieving the SDGs. The study provides vital use cases to contribute to this important research discourse, considering that the WEF resources form the basis for the SDGs, which are essential for the 2030 global agenda.

The present study has generally established that WEF security is closely related to the sustainability of humans, the environment and the economy. Inadequate supply or access to WEF resources can cause several health issues due to poor water quality, an unhealthy diet, and an intermittent lack of energy. Such effects become greater when these variables are combined. It will be beneficial for future work to investigate the following topics to sustainably improve the livelihoods and human and ecosystem health and wellbeing. The following research topics are recommended for future study: (1) the development and optimization of a sustainable and equitable green and blue economy in Africa and (2) the integration of early warning multi-level approach into the governance and management of WEF resources in vulnerable communities.

## 4 Conclusions

The present study used data from questionnaires, focus group discussions, and observations from fieldwork in Narok County and Vhembe District Municipality to evaluate climate change’s explicit or implicit impacts on WEF resources. The questionnaire was well-structured, as most respondents had an opinion about the statements, and only a small percentage of respondents were neutral. The results depicted significant interdependencies between climate and WEF resources. The findings in Narok County and Vhembe District Municipality pointed out localized climate change impacts on the analyzed variables (Appendix A). Undoubtedly, the communities in both study sites have experienced a scarcity of WEF resources due to climate change. The results from the SEM model indicated that water energy and sustainability are affected by climate change. At the same time, climate change directly influences food, confirming findings reported in other literature reports. Furthermore, the results confirmed the existence of synergies in the WEF resources, as reported in the literature, which can be minimized by integrating all processes into a single index to investigate subsystem synergy. Overall, the SEM framework is an effective methodology duly suited to explore interactions between the WEF nexus and the changing climate to advance the WEF nexus research field. Our exploration of WEF resource interactions from the SEM perspective contributes to the body of knowledge of complex nonlinear systems analysis. The WEF nexus is considered a fairly new approach and is still too theoretical for policy- and decisionmakers; hence, its relevance is not well-vested in society. The analysis from the present study provides recommendations for policy- and decisionmakers in scaling up and implementing the WEF nexus approach. To achieve sustainable development within the WEF sectors, this study adopts an integrated approach encompassing political systems, various stakeholders, and role-players working together to define and derive appropriate trade-offs with the WEF resources value chain, especially in the current changing climate. Furthermore, it is recommended to strengthen the integration of the institutions charged with policy and decision making and implementation of WEF nexus approaches. Lastly, it is hoped that the study brings to the fore the important value of wide stakeholder participation, prioritizing citizens to promote knowledge sharing, bottom-up solutions, and conflict resolution.

## Supplementary Material

Supplementary Materials

## Figures and Tables

**Table 1:** Descriptive statistics for the *t*-test and Bartlett test.

Options	*t*-Test	Bartlett Test
Narok	*p*-value < 0.00	*p*-value = 0.00
Vhembe	*p*-value < 0.00	*p*-value = 0.50
Combined	*p*-value < 0.00	*p*-value = 0.00

**Table 2:** Descriptive analysis for multi-regression analysis.

Options	Contrasts	Independent Variable with Significance	*p*-Value	Adjusted r-Squared
Narok	Climate Change	Exposure Nutrition	0.00	0.25
	Sustainable Development		0.000	0.16
	Community Recovery		0.020	0.06
Vhembe	Climate Change	Exposure Nutrition	0.13	0.46
	Sustainable Development		0.68	−0.14
	Community Recovery		0.32	0.22
Combined	Climate Change	Exposure Nutrition	0.00	0.26
	Sustainable Development		0.00	0.15
	Community Recovery		0.01	0.08

**Table 3:** Descriptive analysis for multi-regression analysis for combined constructs.

Predictors	Sust		Water		Energy		Climate		Food	
	Estimates	CI	Estimates	CI	Estimates	CI	Estimates	CI	Estimates	CI
(Intercept)	0.90	−0.30–2.10	1.30*	0.14–2.46	1.50 **	0.53–2.47	3.04 ***	2.36–3.73	0.25	−0.78–1.27
WAT1	0.12	−0.02–0.26			−0.02	−0.13–0.09	0.05	−0.05–0.14	0.26 ***	0.15–0.37
WAT2	−0.14	−0.30–0.03			0.32 ***	0.20–0.44	0.03	−0.08–0.14	−0.12	−0.25–0.01
WAT3	0.11	−0.05–0.27			0.12	−0.01–0.25	−0.04	−0.15–0.07	0.04	−0.09–0.17
ENE1	0.10	−0.12–0.31					−0.07	−0.21–0.07	0.22 *	0.05–0.39
ENE2	−0.11	−0.33–0.10					0.03	−0.12–0.17	−0.14	−0.31–0.03
CLI1	0.20	−0.01–0.42	0.08	−0.12–0.28	−0.06	−0.23–0.11			0.30 ***	0.12–0.47
CLI2	0.15	−0.12–0.42	0.11	−0.14–0.36	−0.09	−0.29–0.12			0.13	−0.08–0.35
CLI3	0.21	−0.05–0.48	−0.07	−0.31–0.18	−0.02	−0.22–0.18			−0.00	−0.21–0.21
MED1			0.04	−0.10–0.17	0.03	−0.08–0.14	0.10 *	0.02–0.19	0.06	−0.05–0.16
MED2			0.04	−0.10–0.17	−0.00	−0.11–0.10	−0.00	−0.09–0.08	−0.02	−0.13–0.09
MED3			−0.05	−0.20–0.11	−0.07	−0.19–0.06	0.11 *	0.00–0.22	0.15 *	0.01–0.28
FOO1			0.38***	0.23–0.54	0.14 *	0.01–0.27	0.21 ***	0.10–0.32		
FOO2			0.00	−0.16–0.16	0.15 *	0.00–0.30	−0.10	−0.22–0.03		
Observations	197		188		208		212		212	
R^2^/R^2^ adjusted	0.178/0.143		0.166/0.129		0.303/0.264		0.223/0.184		0.315/0.278	

* *p* < 0.05

** *p* < 0.01

*** *p* < 0.001.

**Table 4:** Five (5) percent confidence interval for the mediated path.

	Water, Energy, Climate, and Sustainability			Water, Food, Climate, and Sustainability			Energy, Food, Climate, and Sustainability		
Mediation Paths	SD	CI (25%)	CI (97.5%)	SD	CI (25%)	CI (97.5%)	SD	CI (25%)	CI (97.5%)
Water–climate– sustainability	0.04	−0.11	0.02	0.02	−0.07	0.02	-	-	-
Energy–climate– sustainability	0.03	−0.14	−0.01	-	-	-	0.03	−0.12	−0.00
Food–climate– sustainability	-	-	-	0.05	0.08	0.24	0.05	0.07	0.25
Climate– sustainability	0.07	0.32	T0.59	0.06	0.28	0.53	0.07	0.23	0.52

## Data Availability

The raw data supporting the conclusions of this article will be made available by the authors on request.
