# Supplementary material for: Sustainability of the Linkages Between Water–Energy–Food Resources Based on Structural Equation Modeling Under Changing Climate: A Case Study of Narok County (Kenya) and Vhembe District Municipality (South Africa)
Source: Sustainability. Author manuscript; Available in PMC 2025 Mar 15. (PMC7617494; doi:10.3390/su16229689)
Supplement: Supplementary Materials [file EMS203575-supplement-Supplementary_Materials.pdf]

Appendix A

Short Name	Full Name	Question from Survey
wef_dev	Development	Which illustrates that the community experiences scarcity of water, energy, and food resources due to changes in population and urbanization?
Wef_cc	Climate Change	The community/region experiences scarcity of water, energy, and food resources due to changes in climate change hazards.
vul	Vulnerability	The community/region is a high-risk area and is vulnerable to extreme weather due to economic and socio-environmental drivers of change.
exp	Exposure	The community has high exposure and is sensitive to the limited water, energy, and food resources due to economic and socio-environmental drivers of change.
wash	Water, Sanitation, and Hygiene	The community experiences a mortality rate that can be attributed to unsafe water, unsafe sanitation, and lack of hygiene.
nutr	Nutrition	The malnutrition prevalent in the community is associated with the food insecurity among the residents.
a_water	Access to Water	Available and accessible freshwater resources in the community can meet our needs now and in the future.
c_rec	Recovery	Community can recover from economic and socio-environmental disruptions (e.g., famine, floods, high food prices, conflict).

Short Name	Full Name	Question from Survey
p_gov	Government	Weak government institutions impact the wellbeing of the community.
crop_irr	Crop Irrigation	Crops produced through irrigation in the community can meet our needs now and in the future.
a-elec	Access to Electricity	The electricity accessible to the community is enough to use now and in the future.
ac_ecd	Economic Development	Energy produced to support economic growth in the community is enough for our needs now and in the future.
s-food	Food Security	Our access to nutritious and affordable food by the community can meet our needs now and in the future.
